# Impacts of Steel-Slag-Based Silicate Fertilizer on Soil Acidity and Silicon Availability and Metals-Immobilization in a Paddy Soil

**DOI:** 10.1371/journal.pone.0168163

**Published:** 2016-12-14

**Authors:** Dongfeng Ning, Yongchao Liang, Zhandong Liu, Junfu Xiao, Aiwang Duan

**Affiliations:** 1 Ministry of Agriculture Key Laboratory of Crop Water Use and Regulation, Institute of Farmland Irrigation Research, Chinese Academy of Agricultural Sciences, Xinxiang, China; 2 Ministry of Education Key Laboratory of Environment Remediation and Ecological Health, College of Environmental & Resource Sciences, Zhejiang University, Hangzhou, China; INRA, FRANCE

## Abstract

Slag-based silicate fertilizer has been widely used to improve soil silicon- availability and crop productivity. A consecutive early rice-late rice rotation experiment was conducted to test the impacts of steel slag on soil pH, silicon availability, rice growth and metals-immobilization in paddy soil. Our results show that application of slag at a rate above higher or equal to 1 600 mg plant-available SiO_2_ per kg soil increased soil pH, dry weight of rice straw and grain, plant-available Si concentration and Si concentration in rice shoots compared with the control treatment. No significant accumulation of total cadmium (Cd) and lead (Pb) was noted in soil; rather, the exchangeable fraction of Cd significantly decreased. The cadmium concentrations in rice grains decreased significantly compared with the control treatment. In conclusion, application of steel slag reduced soil acidity, increased plant–availability of silicon, promoted rice growth and inhibited Cd transport to rice grain in the soil-plant system.

## Introduction

Rice (*Oryza sativa* L.) is the second most important staple food for more than half of the world’s population [1]. Rice is also a typical Si hyper-accumulating plant species, containing Si up to 10% in shoots on a dry weight basis [2]. Although Si has not been considered as an essential nutrient for plant, there is increasing evidence proved that Si is beneficial for rice growth, development and resistance against abiotic (metal toxicity, salt and drought stress, nutrient imbalance) and biotic stress (plant diseases and insect pests) [3–6]. Rice roots take up Si in the form of silicic acid (H_4_SiO_4_) from the soil solution [2, 5]. In tropical and subtropical areas, plant-available Si is low in these highly-weathered soil owing to heavy desilication-aluminization [7]. Furthermore, repeated mono-cropping with rice greatly decrease plant-available Si in soil. Si deficiency in paddy soil has been recognized as a limiting factor for rice production [2, 8]. Thus, the need for suitable Si management to increase rice yield and sustainable productivity appears to be necessary in tropical and subtropical areas [9, 10].

With the rapid industrialization and economic development in the recent decades, soil heavy metal pollution has been becoming a serious environmental problem in China [11, 12]. The heavy metal contaminated croplands have reached 20 Mha, and most of them are still cultivated [13]. In addition, the area and intensity of paddy soil acidification in the South China have been gradually increasing because of long-term overuse of N fertilizer and acid deposition [14]. Soil acidification increased the mobility and bioavailability of heavy metals [15]. Therefore, it is necessary to find an economical method for remediation of heavy metals contaminated paddy soil.

Steel slag is a byproduct from steel industries which accounts for 15–20% of total steel production [16]. China is the largest producer of steel in the world, huge amounts of steel slag are produced every year in China [17], but only 10% of the steel slag is recycled [18]. Steel slags contain numerous nutrient elements, such as CaO (40–50%), SiO_2_ (10–28%), as well as small amounts of MgO (2.5–10%), Fe_-total_ (17–27%) and MnO (1.5–6%) [19]. Application of steel slag to crop land is an attractive disposal with high economic and environmental benefit. Steel slags have been applied as calcium silicate fertilizer in fields where plant-available silicon is deficient in soil. It is well documented that application of the slag-based silicon fertilizer can not only improve the fertility of degraded paddy soils, but also enhance the growth and yield of rice and the resistance to plant diseases [20–24]. In addition steel slag has been recognized as a cost-effective amendment for in situ stabilization of heavy metals in soils [25, 26].

With 80% of the paddy fields in China being located in the subtropics, it is necessary to demonstrate the effects of steel slag application on rice yield and heavy metal immobilization in the subtropical Chinese paddy fields. Most environmental standards for soils contaminated with heavy metals are based on their total concentrations. However, total concentrations of heavy metals in soil provide very limited information about their chemical behavior and potential fate [27, 28]. The European Community Bureau of Reference (BCR) sequential extraction is considered a useful technique for determining chemical forms of metals in soils which provides information concerning metal potential mobility and plant availability [29–31].

In the present study, a pot experiments were carried out (1) to assess the effect of application rates of steel slag on improving soil acidity and silicon-availability (2) to demonstrate the effects of steel slags on the immobilization of heavy metals in contaminated soil, (3) to test the impacts of slag application on rice productivity and metals accumulation.

## Materials and Methods

### Soil and steel slag material preparation

The soil used was sampled from Qiyang, Hunan province of South China, (no specific permissions were required for soil sampling in this location and the field in this study did not involve endangered or protected species). The soil tested is a paddy soil (anthropogenic soil) derived from red soil (Ultisol). The total Cd concentration is 0.28 mg kg^-1^ Cd, exceeding the Chinese Soil Environmental Quality Standard (0.2 mg kg^-1^ Cd) (GB15618-1995). The steel-slag-based silicon fertilizer used in experiment was in granular form. Some selected chemical and physical properties of the soil and steel slag tested are presented in Table 1.

**Table 1:** Soil basic characteristics and total heavy metal concentrations in the soil and steel slag used in the field experiment.

Item	Soil	Steel slag
pH	5.48	11.99
Organic matter (g kg^-1^)	2.14	nd
Total N (g kg^-1^)	0.89	nd
Available P_2_O_5_ (mg kg^-1^)	7.74	nd
Available K_2_O (mg kg^-1^)	169.4	nd
Available SiO_2_ (mg kg^-1^)	91.6	163000
Available CaO (mg kg^-1^)	/	326000
Cd (mg kg^-1^)	0.474	0.389
Pb (mg kg^-1^)	33.4	3.77
Hg (mg kg^-1^)	0.131	0.219
As (mg kg^-1^)	6.18	1.34
Specific gravity	/	3.92

nd- not detectable (<0.05mg kg^-1^)

### Experimental design

A consecutive pot experiment was conducted in the greenhouse of the Chinese Academy of Agricultural Sciences from March to December 2015. Rice (*Oryza sativa* L. cv. Fengyuanyou 299) used was a hybrid cultivar, characterized by its mid-late maturity. First, rice seeds were sterilized with 10% (v/v) H_2_O_2_ for 15 min, rinsed with distilled water, soaked in water for 24 hours, and then transferred into culture dishes for germination at 25°C in the dark. Two days later, the germinated seeds were placed on a float tray in a controlled environment with a day/night temperature of 25°C (12 h): 25°C (12 h). Each pot was loaded with 5 kg of air-dried and sieved (2 mm) latosol. Five Si treatments with three replicates each were designed. The amounts of Si applied, equivalent to 0.5 M HCl-soluble Si, was 0 (CK), 400 (Si1), 800 (Si2) and 1200 (Si3) 1600 (Si4), 2000 (Si5) mg SiO_2_ per kg soil. The steel slag fertilizer was applied as the basic fertilizer in each season before rice transplanting. First, steel slag fertilizer was incorporated well with 5.0 kg soil in each plastic pot. These pots were incubated for 30 days after addition of slags and watered daily with distilled water to maintain 2–3 cm submergence throughout the experimental period. Thirty days later, basal fertilizers supplied were 0.2 g N kg^-1^ as urea, 52 mg P kg^-1^ as potassium dihydrogen phosphate, and 84 mg K kg^-1^ as potassium sulfate. Two days later, uniform seedlings with three leaves fully expanded were transplanted at two seedlings per pot. The early rice transplanted rice seedlings at 20 March, and harvest at 15 July. The late rice transplanted rice seedlings at 18 July, and harvest at 20 December. During the rice growing period, applied to maintain a 2-cm water layer and no pesticides were applied.

### Plant sampling

Rice plants were harvested at maturity, and separated into stem, leaf, and grain, and then washed thoroughly with distilled water. The dry weight of these tissues was recorded after being oven-dried at 75°C till a constant weight. These tissues were then ground to pass through a 0.5-mm sieve for Si analysis.

### Soil and plant sample collection

Soil samples were collected both before rice transplanting and after rice harvesting in each rice season. Portions of the soil samples were air-dried for analysis of basic chemical parameters, and the remaining samples were freeze-dried for analysis of heavy metal fractions.

### Chemical analysis

Soil pH was determined in 1:2.5 soil/water suspensions using Sartorins PB-10 pH electrode; soil organic matter was measured by wet digestion with K_2_Cr_2_O_7_/H_2_SO_4_; total N was determined by the micro-Kjeldahl method; available P (Olsen P) was extracted by 0.5 M NaHCO_3_ [pH 8.5, soil/NaHCO_3_ ratio of 1:20] and shaken for 0.5 h and determined by molybdenum blue method; available K was extracted by 1.0 M CH_3_COONH_4_ [pH 7.0, soil/CH_3_COONH_4_ ratio of 1:10] and shaken for 0.5 h and determined by AAS; available Si, extracted by 0.25 M citric acid, [soil/(citric acid) ratio of 1:10] and incubated at 40°C for 5 h and analyzed by silicon molybdenum blue spectrophotometry [32, 33].

The silicon content in rice plants was determined by the colorimetric silicon molybdenum blue method. Briefly, 100 mg of plant tissue was mixed with 3 mL of 50% NaOH in a polyethylene tube and covered with a loose-fitting plastic cap and autoclaved at 125°C for 1 h. Silicon in the digested solution was assayed by silicon molybdenum blue spectrophotometry [34, 35].

The available silicon and calcium concentrations in slag were determined following extraction by 0.5 M HCl [slag/(HCl) ratio of 1:50] and shaken for 1 h. Si was analyzed by the colorimetric silicon molybdenum blue method and Ca was analyzed by AAS [36].

Total concentrations of Cd and Pb in soil, or steel slag or plant tissue were digested using the US EPA method 3052. A representative sample of up to 0.5 g was digested in 9 mL of concentrated nitric acid (HNO_3_) and 3 mL of concentrated hydrofluoric acid for 15 minutes in a suitable laboratory microwave system. After the vessel had been cooled, the solution was filtered and diluted to 25 mL with 0.5 M HCl.

The sequential extraction of heavy metal in soil or slag was performed using a three-step procedure recommended by BCR [9], with the details presented in Table 2.

**Table 2:** BCR sequential extraction method of heavy metal in steel slag.

Fraction (0.5 g soil)	Reagent conditions
Exchangeable state (F1)	20 mL of 0.11 M acetic acid (pH 2.8), 16 h continuous shaking under 25°C.
Reducible state (F2)	20 mL of 0.1 M NH_2_OH·HCl (adjusted to pH 2 with HNO_3_), 16 h continuous shaking under 25°C.
Oxidizable state (F3)	5 mL of 30% H_2_O_2_ covered with a watch glass at room temperature for 1 h, then 85°C in a water bath for 1 h, repeat once; add 20 mL of 1M ammonium acetate (adjusted to pH 2 with HNO_3_), 16 h continuous shaking under 2°C.
Residual state (F4)	5 mL HNO_3_+2 mL HF in poly (polytetrafluoroethylene) beakers, heated on a hot plate and evaporated to near dryness.

The concentration of Cd and Pb was determined with ICP-MS, while that of Hg and As was measured with atomic fluorescence spectrophotometer (AFS-2202).

### Statistical analysis

All data in figures and tables were shown as means ± SD of three replicates. A one-way ANOVA was used for statistical analysis and L.S.D. test was adopted to detect the significant difference (*P*<0.05) between the means of different treatments in the same rice season. All statistical analyses were done using SPSS 18.0 and all figures were drawn using Origin 8.0 software.

## Results

### Soil pH and silicon concentration in soil

**Fig 1:**
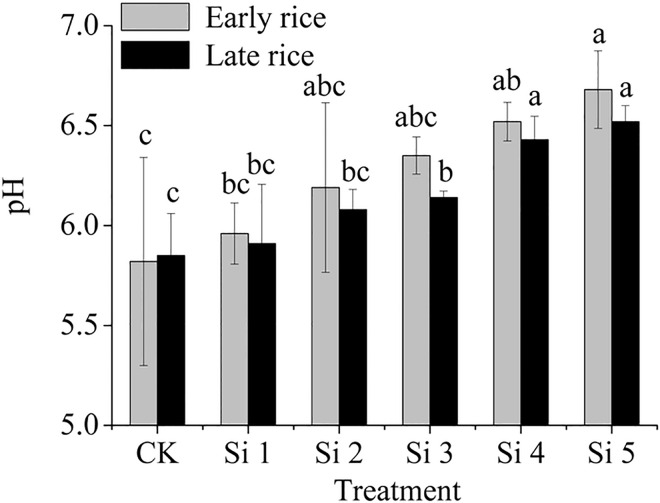
Effect of steel slag fertilizer application on soil pH. Data are means of three replicates. Mean values followed by different letters (a, b, c) in the same season are significantly different (*P<* 0.05).

Soil pH increased with increasing steel slag application rate (Fig 1). Compared with the control treatment, soil pH in the Si4 and Si5 treatments were increased by 0.70 and 0.86 units, respectively, in the early rice season, compared to 0.58 and 0.67 units in the late rice season.

**Fig 2:**
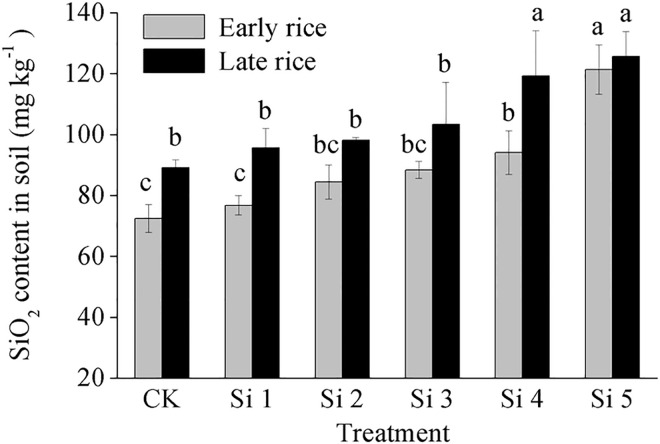
Effect of steel slag fertilizer application on plant available-silicon concentration in soil. Data are means of three replicates. Mean values followed by different letters (a, b, c) in the same season are significantly different (*P*< 0.05).

Plant-available–silicon concentrations in soil tended to increase with increasing steel slag application rate (Fig 2). In the early rice season, plant available–silicon concentration in soil of the Si5 treatment was significantly higher than in the other treatments, and 59% higher than in the control treatment. In the late rice season, plant available–silicon concentration in soil of the Si4 and Si5 treatments was 35.5% and 41.5% higher than in the control treatment, respectively. Except for the Si5 treatment, plant available–silicon concentration in soil of the same treatment was slightly higher in the late rice season than in the early rice season.

### Dry weight and silicon concentration of rice tissues

**Fig 3:**
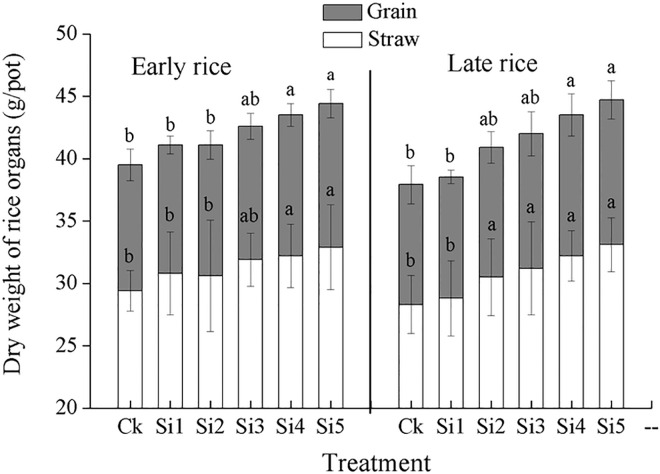
Effect of steel slag fertilizer application on on dry weight of rice organs. Data are means of three replicates. Mean values followed by different letters (a, b, c) in the same season are significantly different (*P*< 0.05).

Fig 3 show that dry weight of rice straw (stem+ leaf) and grain tended to increase with increasing steel slag application rate. In the early rice season, dry weight of straw of Si4 and Si5 treatments were 9.52% and 11.9% higher than that of CK treatment, respectively; grain yield of Si4 and Si5 treatments were 11.9% and 13.9% higher than that of CK treatment, respectively. In the late rice season, application of slag at a rate above higher or equal to 1 200 mg plant-available SiO_2_ per kg soil significantly increased dry weight of straw compared with CK treatment, and equal to 1 600 mg plant-available SiO_2_ per kg soil significantly increased grain yield, with 14.3% and 16.7% higher in the Si4 and Si5 treatments respectively.

**Fig 4:**
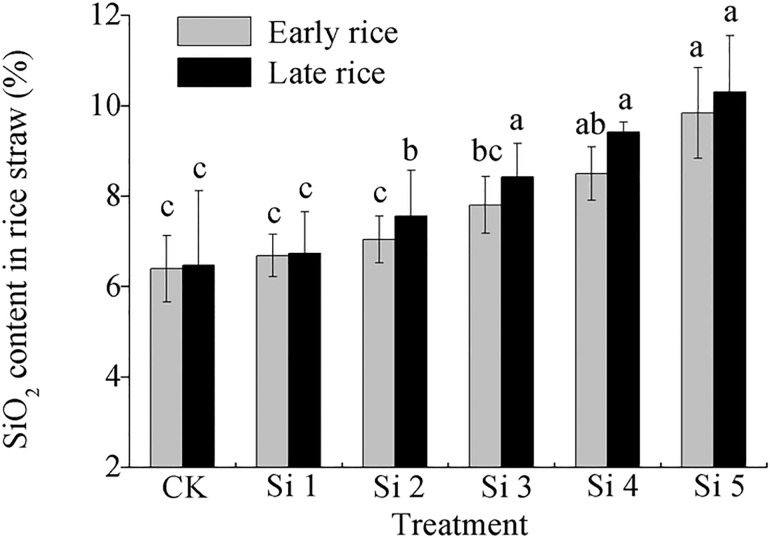
Effect of steel slag fertilizer application on silicon concentration in rice straw. Data are means of three replicates. Mean values followed by different letters (a, b, c) in the same season are significantly different (*P*< 0.05).

Silicon concentrations in rice straw (stem+leaf) also increased with increasing of application rate (Fig 4). In the early rice season, the silicon concentration of rice straw was significantly higher in the Si5 treatment than in the other treatments (except the Si4 treatment), which was 49.9% higher than that of the control treatment. In the late rice season, the silicon concentration of rice straw was significantly higher in all the treatments with slag than in the control treatment, with the silicon concentration being 78.7% and 89.3% higher in the Si4 and Si5 treatments respectively.

### Heavy-metal concentration in soil

**Fig 5:**
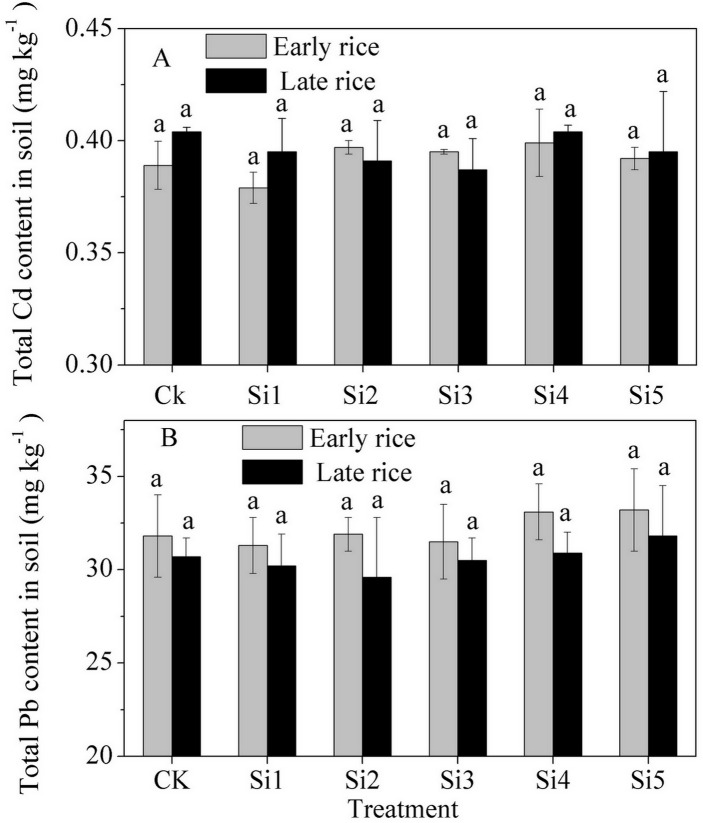
Effect of steel slag fertilizer application on total heavy metal concentration in soil. Data are means of three replicates. Mean values followed by different letters (a, b, c) in the same season are significantly different (P< 0.05).

Table 3 show the BCR concentrations of heavy metals in steel slag. Both Cd and Pb concentrations in steel slag were very low, with the residual fraction accounting for 89.1% and 94.5%, respectively. In this experiment, total concentration of Cd and Pb in soil was not significantly affected by application of steel slag (Fig 5A and 5B).

**Table 3:** BCR extraction concentration of Cr, Cd and Pb in steel slag used in the field experiment.

Metals	BCR extraction			
	F1	F2	F3	F4
Cd (μg kg^-1^)	4.19	1.58	6.96	103.90
Pb (mg kg^-1^)	nd	nd	0.10	1.64

nd-not detectable (<0.05mg kg^-1^)

**Fig 6:**
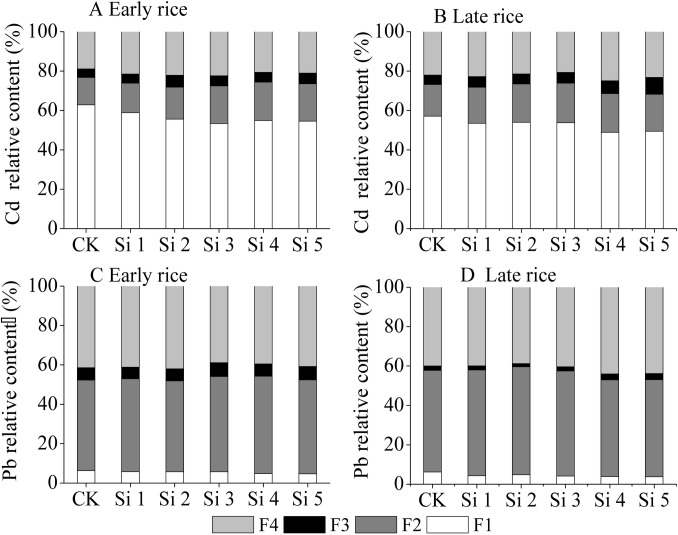
Effect of steel slag fertilizer application on relative content of Cd and Pb in each fraction of soil.

Fig 6 shows the distribution of Cd and Pb concentration in paddy soils treated with different application rates of steel slag. There was a high percentage (50–56%) of exchangeable fraction (F1) of cadmium in soil, while application of steel slag decreased the percentage of the exchangeable fraction (F1), but increased the percentage of the reducible (F2) and residual (F4) fractions (Fig 6A and 6B). In the two rice seasons, when the application rate of steel slag was above 1600 mg plant-available SiO_2_ per kg soil, the F1 fraction was decreased by 12.3–16.3% compared with the control treatment (*P<0*.*05*).

Lead was found to be mainly distributed in the F2 (50%) and F4 (40%) fractions. Application of steel slag decreased the percentage of F1 and increased the percentage of F3, whereas there was little change in F2 or F4 fractions (Fig 6C and 6D).

### Heavy-metal accumulation by rice grain

**Fig 7:**
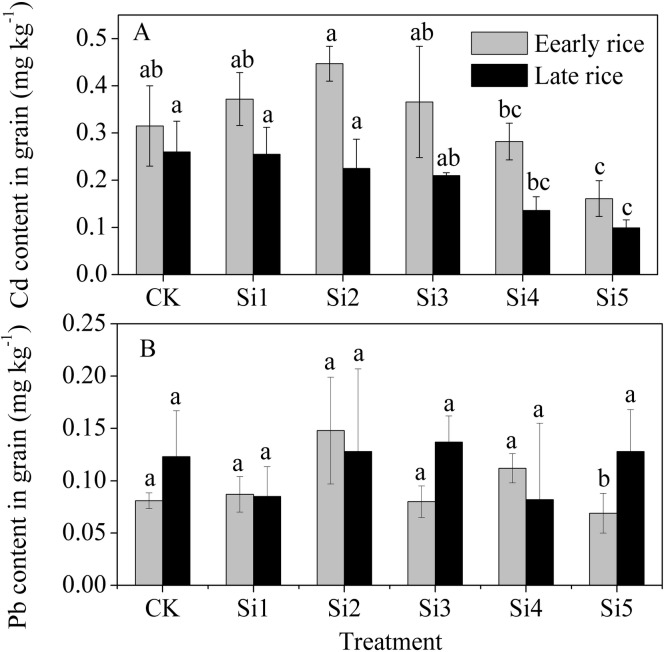
Effect of steel slag fertilizer application on heavy metal uptake by rice grain. Data are means of three replicates. Mean values followed by different letters (a, b, c) in the same season are significantly different (P< 0.05).

Fig 7A and 7B shows the data of Cd and Pb accumulation in the grain of rice grown with different application rates of steel slag. We found that the Cd concentration in rice grain tended to decrease with increasing application rate of steel slag. The Cd concentration of early rice grain and late rice grain was 48% and 63.5% lower in the Si5 treatment than in the control treatment, respectively. In the same treatment with slag, lower Cd concentrations were observed in the late rice than in the early rice. The Cd concentration of early rice grain in the Si5 treatment and that of late rice grain in the Si4 and Si5 treatments were all below the maximum allowable limit level (0.2 mg kg^-1^) (GB 2762–2005).

Application of steel slag had no significant effect on Pb concentration of rice grain. The Pb concentrations of rice grain in all the treatments were found to be below the maximum allowable limit level (0.2 mg kg^-1^) (GB 2762–2005).

## Discussion

### Effect of steel slag on paddy soil pH

Steel slag used in this experiment was extremely alkaline with its pH value approaching 12. When steel slag was applied to paddy soil, such compositions as CaO and MgO dissolved in water with the release of OH^–^, thus increasing soil pH. In the present experiment, original soil pH was 5.48, but soil pH reached 6.43–6.68 when the slag application rate was was higher or equal to 1 600 mg plant-available SiO_2_ per kg soil (Fig 1), which is in line with previous reports [37, 38]. However, it is worth noting that soil pH in the second rice season was not further increased compared with the same treatment in the early rice season. This result suggests that the soil used had a strong buffering capacity at this pH value range (near neutral). The surface of red soil colloids adsorbed large amount of H^+^ and Al^3+^, which contributes to buffer reaction for OH^-1^.

### Effect of steel slag on silicon concentration in soil and rice straw

The initial plant available–silicon concentration in soil was 91.6 mg kg^-1^ SiO_2_. In China, the critical value for available-silicon concentration in paddy soil is 100 mg kg^-1^ SiO_2_, below which positive rice responses to silicate fertilizer can be expected [39]. Applying Si fertilizer to Si–deficient soil is beneficial for sustained rice production [40–41]. Many studies demonstrate that steel slag–based silicate fertilizer was effective in improving both the quality of the degraded paddy soils and the yield and quality of crops [22, 33, 38]. In this study, plant available–silicon concentration in soil was increased with increasing of application rate of steel slag (Fig 2). This increased plant-available silicon concentration in soil mostly was released from slag-fertilizer. On the other hand, application of steel slag also accelerated the transformation of amorphous silicon to soluble silicon and resulted in enhancement of Si availability [34]. Meanwhile, compared with the same treatment in different seasons, plant available–silicon concentration in soil was higher in the late rice season than in the early rice season (except Si5). Two factors could account for this phenomenon. The first factor influencing plant available–silicon concentration may be associated with the different weather conditions in the two seasons. Daily average temperature was about 8°C higher in the late rice season than in the early rice season. The dissolution of Si from the slag used was enhanced by an increase in temperature [36]. The second factor may be related to the residual effect of steel slag applied in the first season. Previous studies showed that only 30% of the available Si in slag could be released during one rice season [21]. However, it is strange to note that plant available–silicon concentration in soil of the Si5 treatment in the late rice season remained unchanged as compared with the same treatment in the early rice season (Fig 2). It was reported that continuous dissolution of Si from steel slag was negatively influenced by the increase of soil solution pH and Ca concentration. The increased pH could enhance the specific adsorption of silicic acid by soil, thus a relatively large percentage of water soluble Si derived from both the soil and the slags could be adsorbed by the soil solid phase [42]. This point of view might explain why continuous application of slag at a huge rate (e.g., 2000 mg plant-available SiO_2_ per kg soil) significantly depressed further dissolution of Si from steel slag.

Rice is a typical Si–accumulating plant species. Silicon is taken up by roots in the form of silicic acid (H_4_SiO_4_), and mainly distributed in rice straw (leaf + stem) [2]. In this experiment, application of slag above higher or equal to 1 600 mg plant-available SiO_2_ per kg soil significantly increased Si concentration in rice straw (Fig 4). As such, accumulation of Si in straw can enhance plant resistance to diseases as silicon concentration in rice straw was negatively correlated with disease severity [43–45]. In the present study, disease incidence and severity (e.g., blast, sheath blight and leaf spot etc.) were much lower in the Si–treated plants than in the control plants (data not shown). So, application of steel slag can provide with silicon nutrition and is beneficial for sustainability of rice production.

### Effect of steel slag on soil heavy metal content and distribution

In this experiment, application of steel slag had no effect on the total Cd concentration in soil (Fig 5A). However, it decreased the percentage of exchangeable fraction and increased that of reducible and residual fraction (Fig 6A and 6B). Two possibilities can be envisaged to explain the decrease of Cd mobility in soils by steel slag. First, soil pH significantly affected Cd solubility in acid soil and the increase of soil pH by alkaline materials decreased Cd availability [46–48]. As pH rose, calcium carbonate and Fe and Mn oxides contained in the steel slag tended to form Cd-carbonate or Cd oxide precipitate compounds [46, 49]. Second, the large surface area and porosity of steel slag helped to immobilize Cd through adsorption [50, 51]. It has been debated whether long-term stability of heavy metals is altered by incorporation of steel slag and similar materials into soil. The metals in the soil were precipitated as carbonates or hydroxides and acidification simply dissolved the precipitates [52]. However, solid-phase diffusion into the lattice of oxides or into the micropores of these oxide materials were considered more stable than pH-moderated sorption of heavy metals in soils [53]. Re-acidification of the soil was a natural soil process, which may result in the liberation of bound metals [47].

Lead was mainly distributed in the Fe/Mn oxide fraction (F2, 50%) and residual fraction (F4, 40%). Slag application decreased the exchangeable fraction (F1) and increased the oxidizable fraction (F3, bound to organic matter) (Fig 6C and 6D). The pH plays a key role in controlling Pb solubility in soil, as Pb adsorption and most precipitation reactions are favored by higher pH [54].

### Effect of steel slag application on plant uptake of heavy metal

Both Cd and Pb concentrations in steel slag were very low, and also mainly present in the form of residual fraction, with their percentage accounting for 89.1% and 94.5%, respectively (Table 3). In the present field experiment, we found that application of steel slag significantly decreased Cd absorption into rice grain (Fig 7A). This decrease, however, could not be only attributed to the pH-rise-caused immobilization of Cd in soil. Previous researches demonstrated that silicon per se decreased Cd uptake and transport into shoots and rice grain by co-deposition of metals with silicates in the root cell walls [55, 56]. Silicon application significantly decreased Cd concentration in the xylem sap and Si-mediated detoxification of Cd in maize plants [46]. Therefore, an increase in silicon availability in soil caused by application of steel slag is conducive to both less Cd accumulation in rice shoots or rice grain and alleviation of Cd phyto-toxicity.

In this experiment, application of steel slag had no significant effect on Pb accumulation in rice grain (Fig 7B). Pb concentrations of rice grain in all the treatments were below the maximum allowable limit value (0.2 mg kg^-1^).

## Conclusion

As a product of waste steel industries, steel slag used in this experiment has been shown great and sustainable potential to reduce Cd accumulation in rice grain. The enhanced soil pH and soil available Si content may contribute to a decrease the available concentration of heavy metals by reducing metal mobility and bonding metals into more stable fractions. Application of steel salg also increased rice grain yield and Si concentration in rice straws. In conclusion, steel slag was an effective amendment for soil acidity adjustment, plant silicon nutrition and stabilization of Cd in acidic soils.

## Supporting Information

S1 Table Effect of steel slag fertilizer application on soil pH. (XLS)

S2 Table Effect of steel slag fertilizer application on plant available-silicon concentration in soil. (XLS)

S3 Table Effect of steel slag fertilizer application on on dry weight of rice organs. (XLS)

S4 Table Effect of steel slag fertilizer application on silicon concentration in rice straw. (XLS)

S5 Table A. Effect of steel slag fertilizer application on total Cd concentration in soil. B. Effect of steel slag fertilizer application on total Pb concentration in soil.
(XLS)

S6 Table A. Effect of steel slag fertilizer application on relative content of Cd in each fraction of soil in early rice. B. Effect of steel slag fertilizer application on relative content of Cd in each fraction of soil in late rice. C. Effect of steel slag fertilizer application on relative content of Pb in each fraction of soil in early rice. D. Effect of steel slag fertilizer application on relative content of Pb in each fraction of soil in late rice
(XLS)

S7 Table A. Effect of steel slag fertilizer application on Cd uptake by rice grain. B. Effect of steel slag fertilizer application on Pb uptake by rice grain.
(XLS)
